# Design optimization and experiment of corn U-shaped fertilization device

**DOI:** 10.1038/s41598-023-36746-5

**Published:** 2023-06-14

**Authors:** Zheng Zuo, Jianguo Zhao, Baozhong Yin, Lixia Li, Hetong Jia, Baochuan Zhang, Zhikai Ma, Jianjun Hao, Zhenyang Wang

**Affiliations:** 1 College of Mechanical and Electrical Engineering, Agricultural University of Hebei, Baoding, 071001 China; 2 College of Plant Protection, Agricultural University of Hebei, Baoding, 071001 China; 3 Institute of Plant Nutrition, Resources and Environment, Beijing Academy of Agriculture and Forestry Sciences, Baoding, 100097 China

**Keywords:** Mechanical engineering, Environmental impact

## Abstract

Aiming at the problems of low utilization rate of corn fertilizer, low precision of fertilization ratio, and time-consuming and laborious topdressing in the later stage, an U-shaped fertilization device with uniform fertilizer mechanism was designed. The device was mainly composed of uniform fertilizer mixing mechanism, fertilizer guide plate and fertilization plate. Compound fertilizer was applied on both sides and slow/controlled release fertilizer was applied at the bottom to form an U-shaped distribution of fertilizer around corn seeds. Through theoretical analysis and calculation, the structural parameters of the fertilization device were determined. Through the simulated soil tank test, the quadratic regression orthogonal rotation combination design was carried out on the main factors affecting the spatial stratification effect of fertilizer. The optimal parameters were obtained as follows: the stirring speed of the stirring structure was 300 r/min, the bending angle of the fertilization tube was 165°, and the operating speed of the fertilization device was 3 km/h. The results of bench verification test showed that under the optimized stirring speed and bending angle, the fertilizer particles were stirred evenly, and the average values of fertilizer flowing out of the fertilization tubes on both sides were 299.5 g and 297.4 g, respectively. The average fertilizer amounts of the three fertilizer outlets were 200.4 g, 203.2 g and 197.7 g, respectively, which met the agronomic requirements of 1:1:1 fertilization, and the variation coefficients of fertilizer amounts on both sides of the fertilizer pipe and each layer were less than 0.1% and 0.4%, respectively. The simulation results of the optimized U-shaped fertilization device can achieve the expected U-shaped fertilization effect around corn seeds. The results of field experiment showed that the U-shaped fertilization device could realize the U-shaped proportional application of fertilizer in soil. The distance between the upper end of fertilization on both sides and the distance between the base fertilizer and the surface were 87.3–95.2 mm and 197.8–206.0 mm, respectively. The transverse distance between the fertilizers on both sides was 84.3–99.4 mm, and the error with the designed theoretical fertilization was within 10 mm. Compared with the traditional side fertilization method, the number of corn roots increased by 5–6, the root length increased by 30–40 mm, and the yield increased by 9.9–14.8%.

## Introduction

Corn occupies an important position in the global food system^1^, and corn is also a fertilizer-loving crop^2^. In 2022, China’s corn planting area is 45.88 million hectares. According to the traditional 40 kg/mu fertilization calculation, the fertilizer consumed is as high as 27.53 million tons^3^. By the end of 2020, the fertilizer utilization rate of the three major grain crops in China was 40.2%, which was far lower than the level of 60% in developed countries^4^. Most fertilizers have problems such as volatilization, leaching, and low crop absorption and utilization. Therefore, the reasonable, scientific, and efficient fertilization is of great significance for increasing corn yield, responding to the core goal of “one control and two reductions” in the country^5^, reducing human labor intensity, and improving fertilizer utilization.

In the traditional corn fertilization operation, it is mainly based on staged fertilization^6^. Generally, the combination of side basal fertilizer and late topdressing is adopted^7^. In this way, not only the amount of fertilizer is large and the labor intensity is high, but also the fertilizer utilization efficiency is low, which is difficult to meet the needs of agricultural production in China. In order to improve the traditional fertilization methods, many scholars have studied the full-layer proportional fertilization technology and the corresponding devices.

The full-layer proportional fertilization technology mainly applies the fertilizer required during the growth period of corn to the soil in a one-time proportional manner, which not only improves the utilization rate of fertilizer, but also reduces the process of staged fertilization operation, so as to achieve cost-saving and efficiency-increasing^8^. The literatures basically studied the effect of one-time proportional fertilization. Experiments showed that the corn yield under one-time proportional fertilization was greater than that of traditional staged fertilization, implied that the technology could replace the traditional fertilization method^9^.

In order to effectively implement the full-layer proportional fertilization, Liu et al.^10^ designed a corn subsoiling full-layer fertilization shovel, which applied the fertilizer to the soil in a way that the upper was less and the lower was more. At the same time, it could loosen the deep soil and provided a good growth environment for the development of corn seeds. Zhao et al.^11^ designed a no-tillage planter with deep application of base fertilizer in lateral position and vertical split application of mouth fertilizer, which met the requirements of vertical and lateral distance of seed fertilizer, stability of machine operation and precision of fertilization and sowing. Wang et al.^12^ designed a fertilizer rate adjustable stratified fertilizer applicator. By adjusting the installation angle of the stratified fertilizer applicator and the working length of the fertilizer piece, the fertilizer ratio in each layer was adjusted. The above- designed fertilization devices were effective to fertilize on one side of corn, which was beneficial to promote the growth of the unilateral root system. Reference^13^ showed that unilateral fertilization, plant roots were concentrated to one side, in the water shortage, the contradiction between water was more acute, so that a variety of nutrients were not fully absorbed. References^14^ pointed out that the fertilization on both sides or around the crop increased the fertilizer utilization rate and grain yield, and meanwhile made the plant roots grow on both sides or around, and improved the lodging resistance of plants.

In order to improve the distribution of fertilizer around corn seeds and promote the comprehensive and effective growth of corn roots, an U-shaped full-layer proportional fertilization device was designed. The discrete element method (simulation) was used to study the movement of fertilizer particles in the device and the main factors of fertilizer ratio. The fertilizer ratio of the device and the effect of full-layer proportional fertilization were verified by bench test and field test.

## Methods

### Structure and working principle of U-shape full layer proportional fertilization device

#### Fertilization device structure

**Figure 1:**
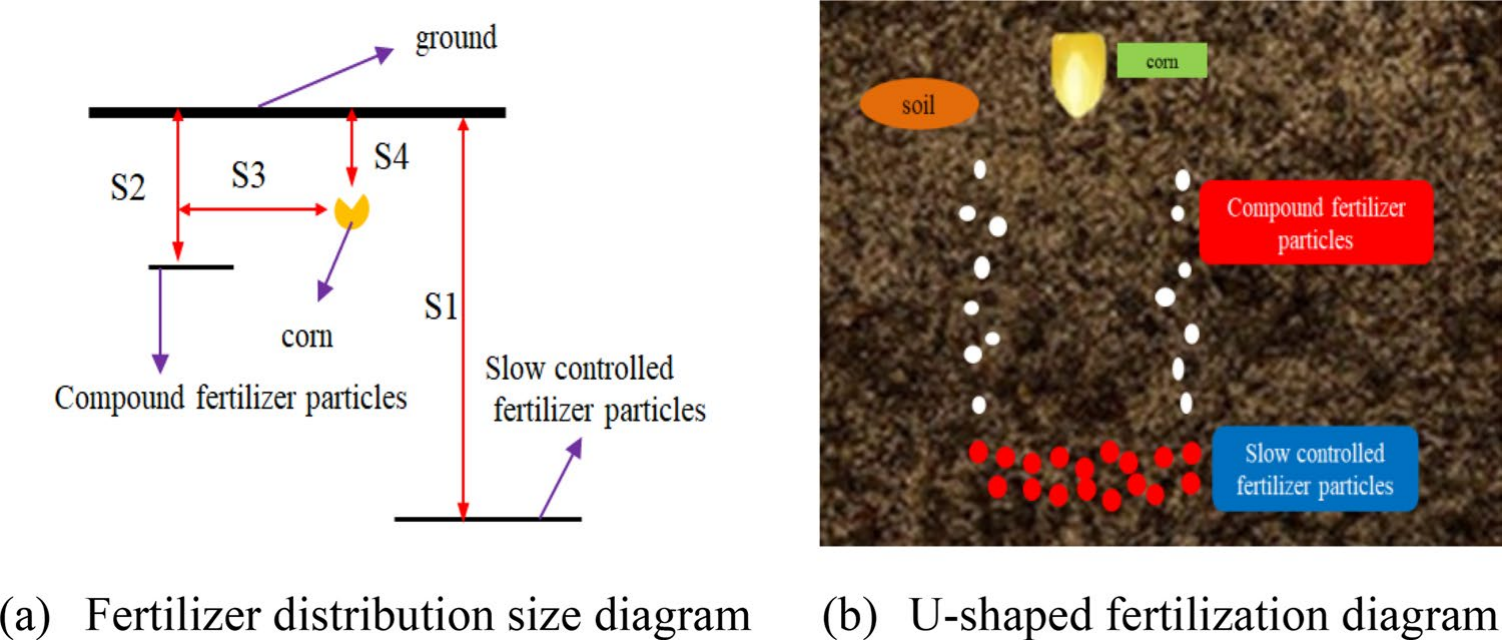
U-shaped fertilization diagram and fertilizer size distribution.

Reference^2^ showed that deep application of fertilizer around or at the bottom of seeds could not only improve the fertilizer efficiency, but also reduce the workload and improve the working efficiency of machinery. Literature^9^ studies have shown that deep fertilization is conducive to increasing fertilizer yield. When seed fertilizer and base fertilizer are applied to the soil at a ratio of 3:7, the yield of crops is the best, which will not cause seed burning and ensure the nutrient supply during the seed growth cycle. Therefore, an U-shaped full-layer proportional fertilization device is designed in this paper. The device can apply the compound fertilizer and slow-controlled fertilizer required for the growth cycle of corn to the soil in a one-time proportional manner, and the fertilizer in the soil presents an U-shaped distribution relative to the corn seed (Fig. 1). The depth of fertilization S1 is 200 mm, and the top of the full-layer fertilization on both sides is 90 mm from the surface depth S2^15^, and the bottom of the full-layer fertilization is consistent with the deep fertilization. The distance S3 between the two sides of the full-layer fertilization and the corn are 50 mm, and the depth of the corn seed from the ground S4 is 30–50 mm.

**Figure 2:**
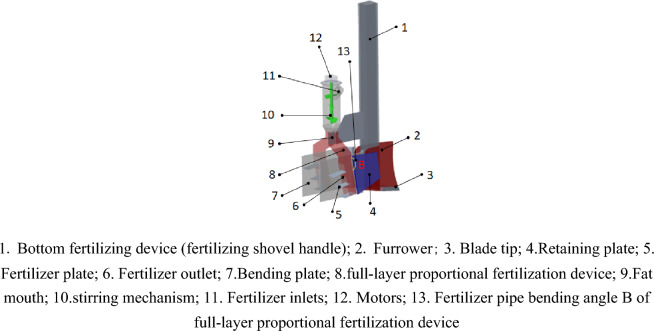
U-shaped full-layer proportional fertilization device structure diagram.

The U-shaped full-layer proportional fertilization device is a key component to realize the U-shaped full-layer proportional fertilization. It is mainly composed of a mixing mechanism, a gear mechanism, a fertilizer distribution mechanism, a deep fertilization mechanism, a furrow opener, and a fertilization plate. The structure is shown in Fig. 2.

#### Working principle

The slow-controlled fertilizer is directly applied to the soil where the opener operates through the fertilizer shovel handle 1; the compound fertilizer enters the device with stirring structure 10 through the fertilizer inlet 11. Under the action of gravity, the motor drives the stirring mechanism 10 to rotate, and the falling fertilizer particles are evenly dispersed. Finally, at the fertilizer inlet 9, the scattered fertilizer is divided into two parts, which are respectively entered into the full-layer proportional fertilization device 8 on both sides. Under the action of the diversion of the fertilizer guide plates 5, the fertilizer is transported to the loose soil of the retaining plate 4. Under the action of soil reflux and the effect of the bending plate 7 blocking the fertilizer on both sides to the center, the fertilizer forms an U-shaped fertilization state in the soil.

### Theoretical analysis of key components

#### Theoretical analysis of stirring structure

**Figure 3:**
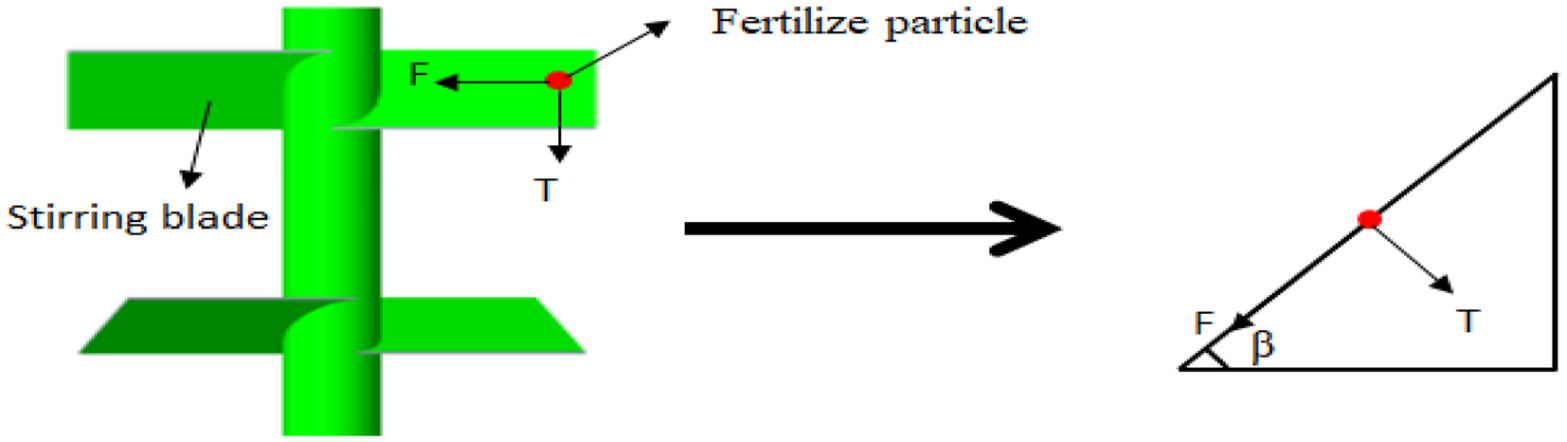
Force analysis of stirring blade.

The length of the stirring shaft of the stirring structure is designed to be 160 mm, and the stirring blades are designed to be plate-like blades that maintain 45° with the horizontal plane. In order to stir more evenly, a total of three blades are designed, each having two blades, and the projection angle of the six blades is 60°. The length of the blade is consistent with the inner diameter of the outer shell of the stirring mechanism. The length of the stirring blade is designed to be 23 mm. Reference^16^, fertilization 600 kg/hm^2^, fertilization pipe diameter is set to 30 mm, fertilizer in the fertilizer pipe circulation is the best. Therefore, the stirring structure is the main force frame in the process of fertilizer falling. The design of the stirring structure mainly considers the strength of the shaft. The force of each blade of the stirring structure is mainly divided into two parts: one is the component load T of the material gravity acting on the blade surface; the second is the shear resistance and friction force F during the blade movement. The force analysis of blade is shown in Fig. 3.

In the process of operation, the stirring center shaft needs to overcome the friction torque *M*_*f*_ generated by the friction between the blade and the material and the driving torque *M*_*t*_ generated by the component load of the material gravity acting on the blade surface when the material rotates *M*_*t*_. Among them $T = \rho gh\cos \beta$,$F = \mu \rho gh\sin \beta$, the integral method is used to calculate the torque of the spindle^17^:

1 $$M_{{\text{f}}} = \int_{0}^{l} {\mu \rho } gh\sin \beta \cos \beta bxdx$$

promoting moment *M*_*t*_

2 $${\text{M}}_{t} = \int_{0}^{l} \rho gh\sin \beta \cos \beta bxdx$$

The calculation formula of total torque M of each blade is:

3 $$\begin{gathered} M = M_{{\text{f}}} { + }M_{t} = \int_{0}^{l} \rho gh(1 + \mu )\sin \beta \cos \beta bxdx \hfill \\ = \frac{{\rho gh(1 + \mu )bl^{2} }}{2} \hfill \\ \end{gathered}$$

where: $\rho$-material density, average 1328 kg/m^3^; $g$-gravity acceleration, 9.8 N / kg; $l$- leaf length, m;$b$-width of central section of blade, m; $h$- material height on blade, m; $\mu$- friction coefficient between material and blade, dynamic friction coefficient is 0.01; $\beta$- the angle between the stirring blade and the horizontal plane after differentiation, °; $x$- integral variable in the length direction of blade.

In terms of safety, the material on the stirring blade is the diameter of three granular fertilizers, that is, h is 0.0105 m. At this time, the device adopts a rectangular interface, 0.001 m, and the length of the blade is 0.025 m. Each parameter into the formula, the blade is calculated by the moment = 8.3 × 10^–5^ In order to ensure the safety and reliability of the design, join the safety factor, material 45 #, stirring shaft minimum diameter button strength to calculate:

4 $$\tau { = }\frac{\psi T}{{W_{T} }} \le [\tau_{T} ]$$

where: $\tau_{T}$ -allow torsional shear stress, $MPa$; $\psi$- safety factor, take 1; $\tau$- torsional shear stress, $MPa$; $T$- the torque on the shaft, $N\;{\text{mm}}$;

Hollow shaft torsion section coefficient $W_{T}$ calculation formula is:

5 $$W_{T} = \frac{{\pi D^{3} }}{16}$$

where: $W_{T}$- the torsional section coefficient of the shaft, mm^3^; $D$- outer diameter of stirring shaft, mm. Calculated $D$ ≥ 14 mm, rounded, take $D$ 14 mm.

#### Force analysis of retaining plate

**Figure 4:**
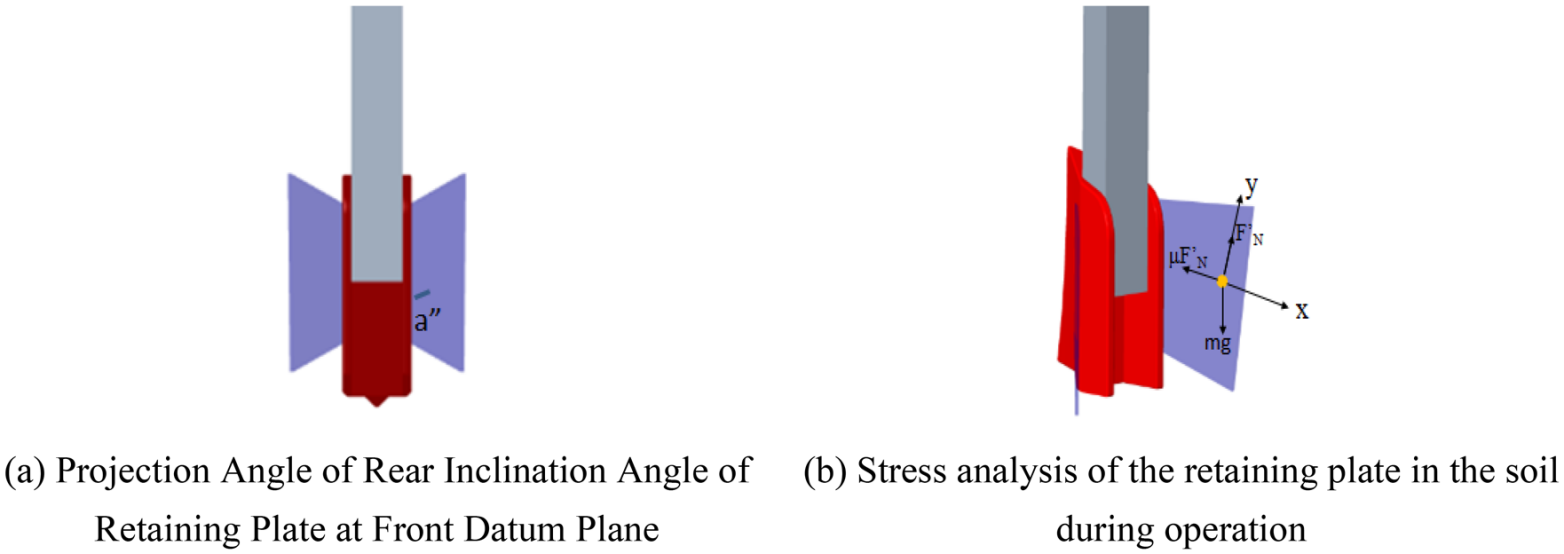
Rear inclination of the retaining plate and force analysis of the working retaining plate.

When the fertilization device is working, the soil above the retaining plate moves relative to the retaining plate. The retaining plate is cut open by facing the datum plane. The soil particles above the retaining plate are taken as the research objects. The movement is shown in Fig. 4. The differential equation of motion of the soil particles is^18^:

6 $$m\frac{{d^{2} x}}{{dt^{2} }} = mg\cos a^{\prime\prime} - \mu F^{\prime}_{N}$$

7 $$m\frac{{d^{2} y}}{{dt^{2} }} = 0$$

Quorum

8 $$F^{\prime}_{N} = mg\sin a^{\prime\prime}$$

Arranged

9 $$\frac{{dv_{x} }}{dt} = g\cos a^{\prime\prime} - \mu g\cos a^{\prime\prime}$$

Pair (9) integral, get

10 $$v_{x} = - gt\sin a^{\prime\prime} - \mu gt\cos a^{\prime\prime} + C_{1}$$

Arranged

11 $$\frac{dx}{{dt}} = - gt\sin a^{\prime\prime} - \mu gt\cos a^{\prime\prime} + C_{1}$$

Pair (11) integral, get

12 $$x = - gt^{2} \cos a^{\prime\prime} + \mu gt^{2} \sin a^{\prime\prime} + C_{1} t + C_{2}$$

where: $m$ -soil particle mass, kg. $g$-gravity acceleration, m/s^2^. $a^{\prime\prime}$-the projection angle of the back-dip angle of the retaining plate at the reference plane, (°). $F^{\prime}_{N}$—soil supported by retaining plate, N. $v_{x}$- velocity of soil particles along X axis, m /s. $C_{1}$, $C_{2}$- motion constants of soil particles at this time, $x$—displacement of soil along X axis, m. $t$-time, s.

It can be seen from Eq. (12) that the movement of soil particles above the retaining plate is affected by the projection angle a″ of the backward inclination angle a of the retaining plate on the facing datum plane. Therefore, the backward inclination angle a of the retaining plate will affect the movement of soil particles, which in turn affect the effect of fertilization. In order to minimize the back bending angle of the retaining plate, the retaining plate is welded together with the fertilizing pipe to minimize the inclination angle of the retaining plate.

### Simulation analysis of fertilization process

The full-layer proportional fertilization mechanism on both sides of the fertilization device has an important influence on the effect of U-shaped fertilization. The simulation model of U-shaped full-layer spatial fertilization device was established by three-dimensional drawing software and simulation software, and the simulation test was carried out. The stirring speed of the stirring structure, the bending angle of the full-layer proportional fertilization device and the operating speed of the U-shaped spatial fertilization device were used as reference factors to optimize the structure and working parameters of the U-shaped spatial fertilization device.

#### Building a simulation model

The discrete element method is used to simulate and analyze the movement process of fertilizer in the fertilization device. The Stanley compound fertilizer is selected as the test material, and its physical characteristic parameters are measured by experiments and referring to the relevant literature^12^. The fertilizer belongs to the round-like particles, and its spherical rate is as high as 90%. Therefore, the simulation model can use the spherical instead of the fertilizer particles. The density of the fertilizer particles is set to 1328 kg/m^3^, the equivalent diameter is 3.2 mm, the Poisson’s ratio is 0.25, and the shear modulus is 1.0 × 10^7^ pa. The variable parameters of the fertilizer particles and the fertilization device model are shown in Table 1.

**Table 1:** Variable parameters of fertilizer particle and fertilization device model.

Parameters	Fertilizer particle	Fertilization device
Poisson 's ratio	0.25	0.30
Density / ( kg/m^3^)	1328	7800
Shear modulus/pa	1.0 × 10^7^	7.0 × 10^10^
Collision restitution coefficient	0.095	0.590
Static friction factors	0.250	0.312
Rolling friction factors	0.1	0.01

#### Single factor test

According to the analysis, the main influencing factors affecting the movement of fertilizer particles in the U-shaped space fertilization device are the stirring speed A of the stirring structure, the bending angle B of the full-layer space proportional fertilization device and the operating speed C of the U-shaped space fertilization device. The single factor simulation test was carried out on the working parameters of the fertilization device. The particle factory of Polygon is set, the generation rate is 500 particles/s, the falling speed is 23 cm/s, the total simulation time is set to 10 s, the Rayleigh time step is 20%, and the data recording interval is 0.1 s^19^.

##### Stirring speed A single factor test

**Figure 5:**
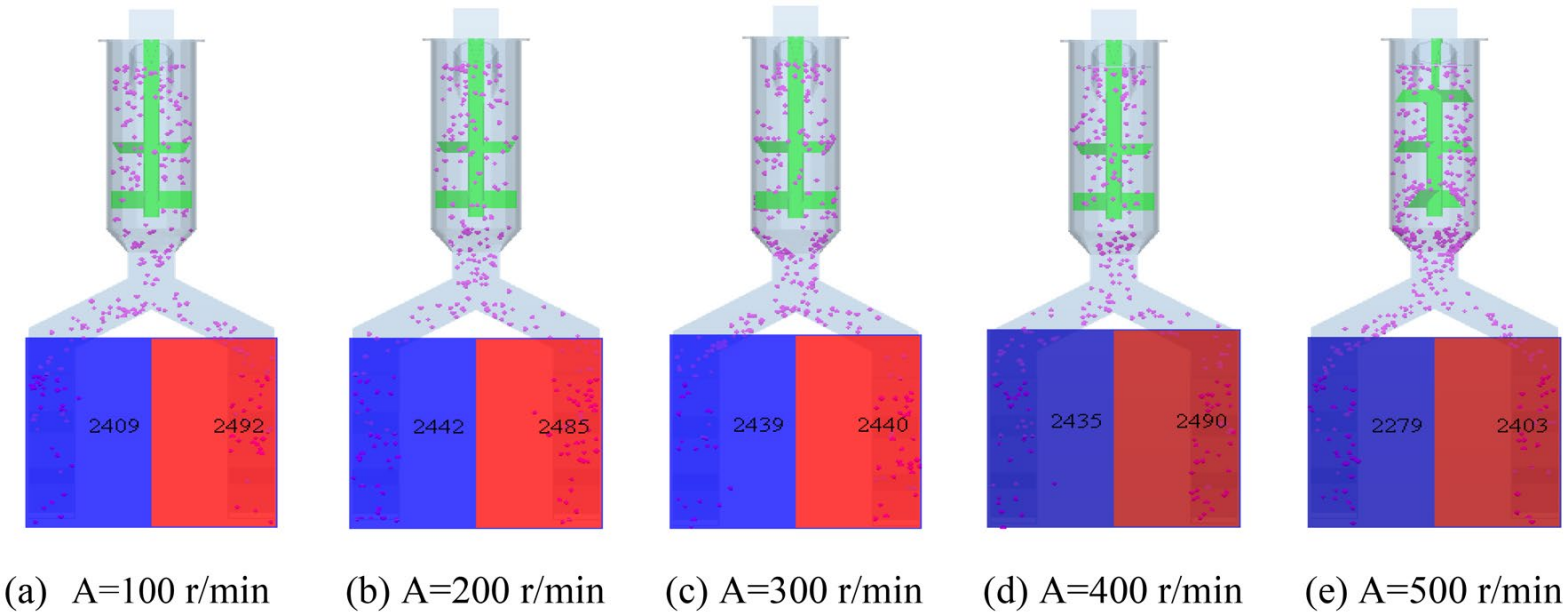
Detection effect of stirring structure.

Set the speed range of the stirring speed A of the stirring structure to 100–500 r/min and divide it by 100 r/min. The simulation is completed and the stirring effect of the stirring structure is detected by using the post-processing of the simulation software, and the detection effect is shown in Fig. 5.

##### Single factor test of bending angle B

**Figure 6:**
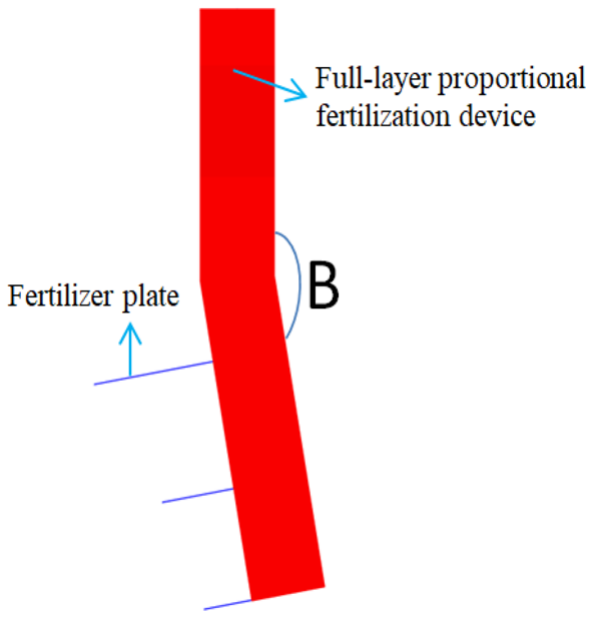
Bending angle B.

**Figure 7:**
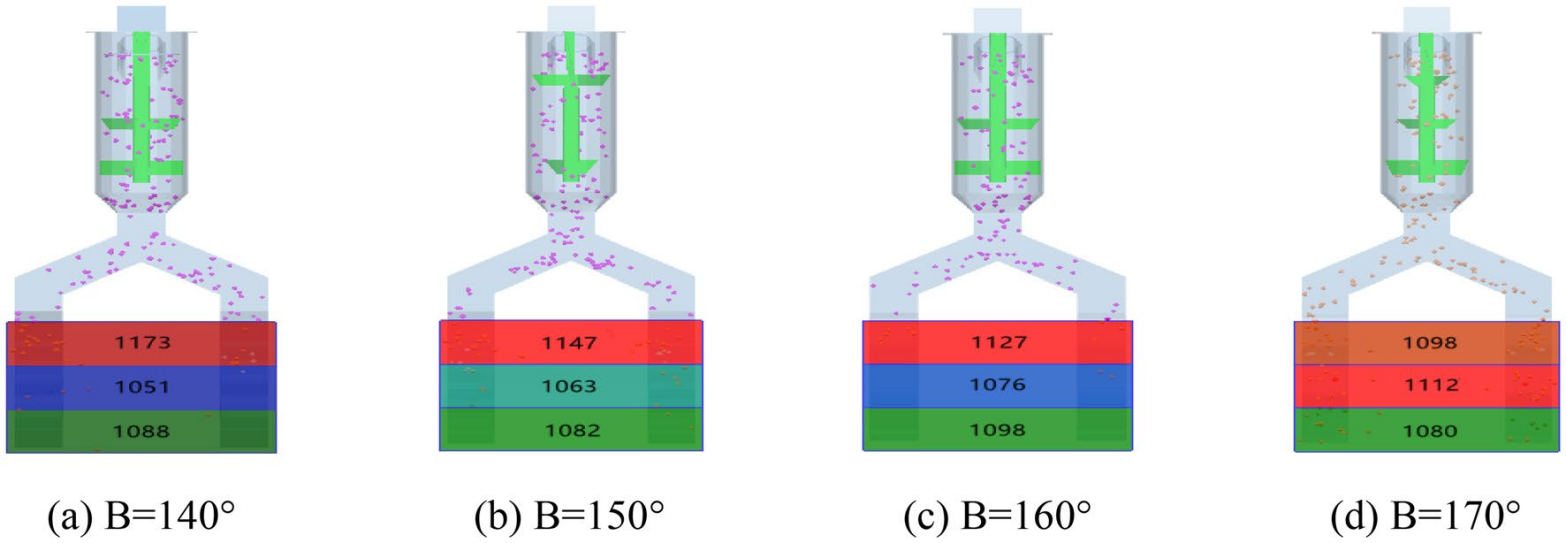
Fertilization amount at different bending angles.

The simulation software was used to carry out the single factor experiment on the bending angle B at the fertilizer pipe under the full-layer proportional fertilization mechanism, and the bending angle B was shown in Fig. 6. The bending angle of 180° was first discharged. Because the fertilizer particles were easily dropped to the bottom of the fertilizer pipe under the action of gravity, the full-layer proportional fertilization could not be realized. Therefore, the bending angle is set to 170°, 160°, 150°, 140°, and the drawn three-dimensional graphics are imported into the simulation software for simulation. The simulation results are shown in Fig. 7.

##### Determination of single factor range of operating speed C

During the operation of the U-shaped full-layer proportional fertilization device, the soil returning speed of each layer of fertilizer depth is one of the key factors affecting the operation effect of full-layer fertilization. After the shovel tip and the retaining plate of the U-shaped full-layer proportional fertilizer device cut the soil, the soil falls by its own gravity to complete the soil returning operation. During the fertilization process, as the U-shaped full-layer fertilization device advances, the shovel tip and the retaining plate lift the broken soil and continue to move backward under inertia. When the running speed increases, the movement distance between the broken soil and the U-shaped full-layer fertilization device is larger, the longer the soil returning time is, and the soil returning is not timely after fertilization, affecting the U-shaped fertilization effect. Therefore, the fertilization speed is also an important factor affecting the effect of layered fertilization. Reference^6^, the fertilization shovel operation speed range is 2–4 km/h.

### Quadratic orthogonal rotation combination design test

#### Simulation test design

In order to study the influence of various factors on the movement of fertilizer particles in the full-layer proportional fertilization device, the mechanism parameters of the U-shaped full-layer fertilization device were optimized. The stirring speed of the stirring structure, the bending angle at the fertilization pipe of the full-layer proportional fertilization device and the operation speed of the fertilization shovel were used as test indicators. The quadratic orthogonal rotation combination design test was carried out to study the influence of various test factors on the amount of fertilizer discharged from the fertilizer outlet, and to optimize the structure and working parameters of the U-shaped fertilization shovel. The horizontal value range of each test factor is 200–400 r/min. The bending angle of the fertilization tube at the full-layer proportional fertilization device is 160–170°; fertilization shovel professional speeds 2–4 km/h. The experimental factors are coded as shown in Table 2.

**Table 2:** Test factor coding.

Coding	Factor		
	Stirring speed A (r/min)	Folding angle B (°)	Operating speed C (Km/h)
1	200	160	2
0	300	165	3
− 1	400	170	4

According to Reference^20^, soil particles are generally spherical, nuclear, columnar three shapes. The smaller the particle size of the soil particles, the better, but the simulation time is longer. In order to improve the calculation efficiency and save time, the spherical soil is represented by a sphere with a diameter of 6 mm. The nuclear soil particles are composed of spheres with a length of 12 mm and a width of 6 mm. The columnar soil is composed of three 6 mm spheres. The simulation parameters mainly involve two sets of parameters, as shown in Tables 3 and 4. Since the fertilizer particles are spherical particles and there is no adhesion on the surface of the particles, the Hertz-Mindlin (no-slip) contact model is used between the fertilizer particles^6^.

**Table 3:** Material parameters in simulation software.

Materials	Density (kg/m^3^)	Poisson 's ratio	Shear modulus (MPa)
Compound fertilizer particles Slow controlled fertilizer particles	1328 1564	0.25 0.25	1.0 × 10^7^ 1.0 × 10^7^
Soil particles	2500	0.3	1.0 × 10^6^
Fertilization device	7800	0.3	7.0 × 10^10^

**Table 4:** Contact parameters between materials in simulation software.

Parameters	Restitution coefficient	Coefficient of static friction	Coefficient of kinetic friction
Compound fertilizer particles Slow controlled fertilizer particles	0.307 0.380	0.372 0.600	0.123 0.300
Fertilization device and compound fertilizer particles	0.590	0.312	0.010
Soil particles	0.200	0.400	0.300
Fertilization device and soil particles	0.300	0.500	0.050
Compound fertilizer particles and soil particles	0.020	1.250	1.240
Compound Fertilizer and Slow Control Fertilizer Granules	0.380	0.600	0.300
Slow Controlled Fertilizer Particles and Soil Particles	0.410	0.400	0.020
Slow controlled release fertilizer granules and fertilization device	0.370	0.500	0.030

#### Soil model construction

**Figure 8:**
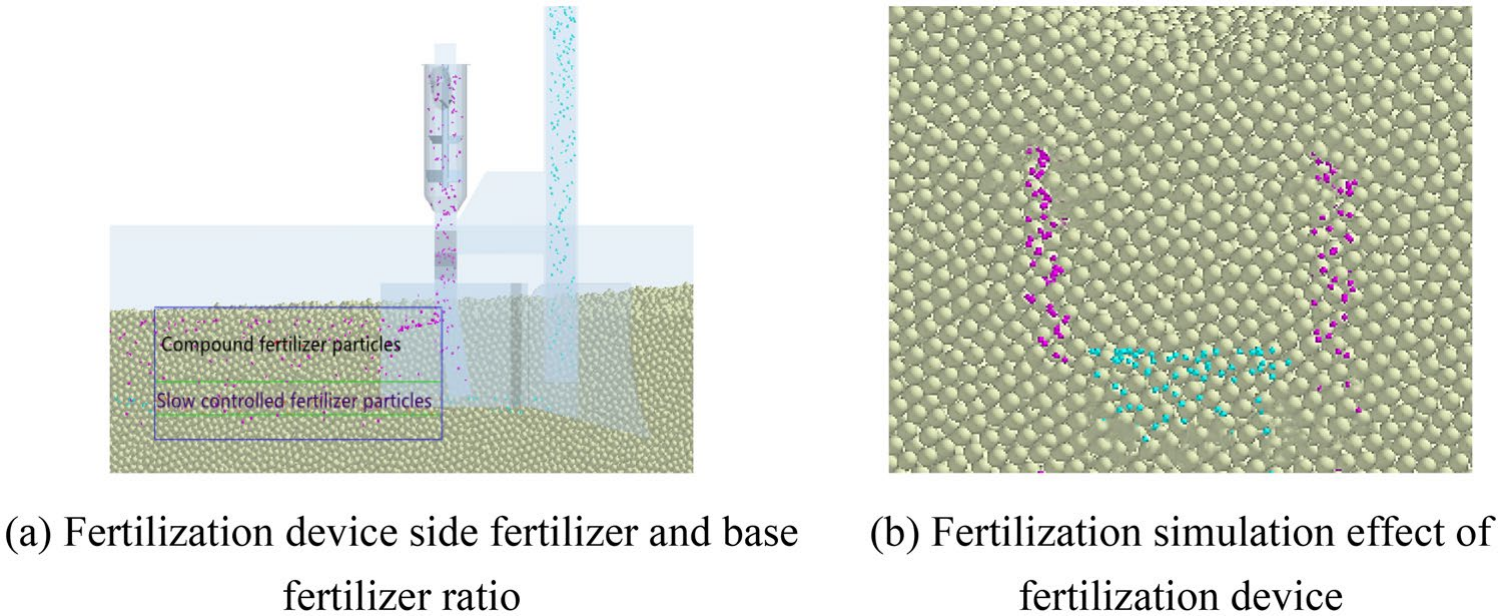
Simulation effect of fertilizing device in soil bin.

The fertilizer used in this experiment is shidanli compound fertilizer, in which N 15%, P_2_O_5_ 15%, K_2_O 15%. At the same time, according to relevant literature, the soil in the experimental area (North China Plain) is silty light loam, with organic matter 16.7 g/kg, total nitrogen 1.1 g/kg, NH4^+^–N 12.1 mg/kg, NO_3_–N 31.6 mg/kg, available phosphorus 14.8 mg/kg, available potassium 174.0 mg/kg^21^. Through the construction of soil model and the selection of contact model, the rectangular virtual soil trough model with the length, width and height of 600 mm, 400 mm and 400 mm, respectively, is established. In the reference^20^, when establishing the soil bin, the number of spherical, nuclear and columnar particles in the soil particles is stacked in the ratio of 1:1:1.While the contact model between the soil bin particles is set to Hertz-Mindlin with JKR V2, and the contact model parameter is set to 9.04 J/m^2^^22^. The simulation effect of the fertilization device in the soil bin is shown in Fig. 8.

According to the amount of fertilizer output from each layer of fertilizer outlet in the soil tank simulation, 17 sets of simulation tests were carried out. Three factors were selected for the simulation model, namely, the stirring speed of the stirring structure, the bending angle of the fertilization tube on both sides, and the fertilization speed of the fertilization device. Each factor was taken at three levels, and the three-factor three-level quadratic regression orthogonal rotation combination designs was carried out. The test plan and results are shown in Table 5.

**Table 5:** Test plan and results.

Numbering	Stirring speed A (r/min)	Folding angle B (°)	Operating speed C (km/h)	Upper fertilizer output M_1_ (g)	Middle fertilizer amount M_2_ (g)
1	400.00	170.00	3.00	180	179
2	200.00	170.00	3.00	188	184
3	400.00	160.00	3.00	183	190
4	200.00	160.00	3.00	240	232
5	400.00	165.00	2.00	193	210
6	200.00	165.00	2.00	213	211
7	400.00	165.00	4.00	187	184
8	200.00	165.00	4.00	214	218
9	300.00	170.00	2.00	207	203
10	300.00	160.00	2.00	240	234
11	300.00	170.00	4.00	189	193
12	300.00	160.00	4.00	230	237
13	300.00	165.00	3.00	203	199
14	300.00	165.00	3.00	197	202
15	300.00	165.00	3.00	213	215
16	300.00	165.00	3.00	205	201
17	300.00	165.00	3.00	198	193

#### Response surface analysis

**Figure 9:**
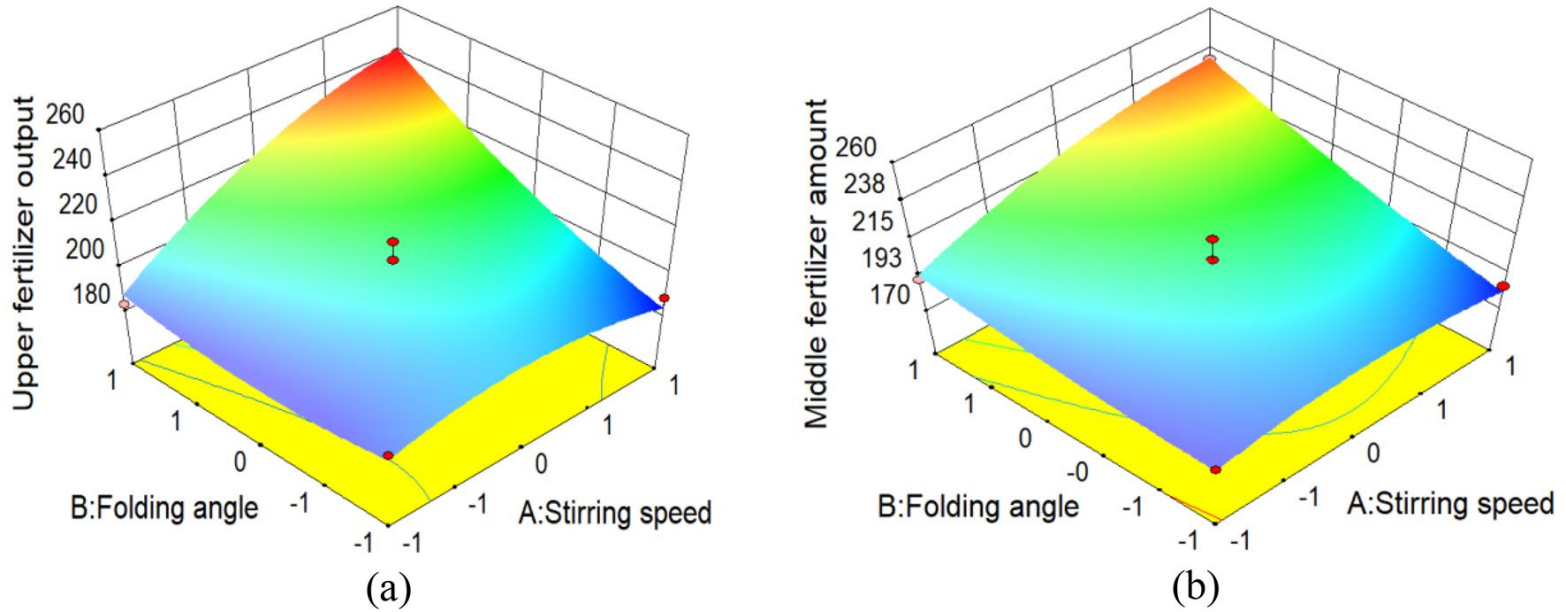
Two-factor response surface curve of fertilization amount.

In order to analyze the relationship between various factors and test indicators, the Design-Expert software was used to analyze and process the experimental data, and the interaction between the stirring speed of the stirring structure, the bending angle of the fertilizer pipe, and the operating speed of the fertilizer shovel was obtained. The effect response surface of the amount of fertilizer applied to the upper and middle fertilizer outlets are shown in Fig. 9.

According to the previous analysis, the upper, middle and lower parts of the whole layer fertilization on both sides were1:1:1, which could effectively improve the fertilizer utilization rate. In the simulation, the total amount of particles generated by the particle factory was 600 g. When the fertilization amount of the upper and middle layers is 200 g, it can meet the 1:1:1 fertilization ratio distribution of the upper, middle and lower parts. Therefore, the fertilization amount of the upper and middle layers is 200 g as the target fertilizer amount for analysis.

### Bench verification test

#### Test conditions and evaluation indexes

**Figure 10:**
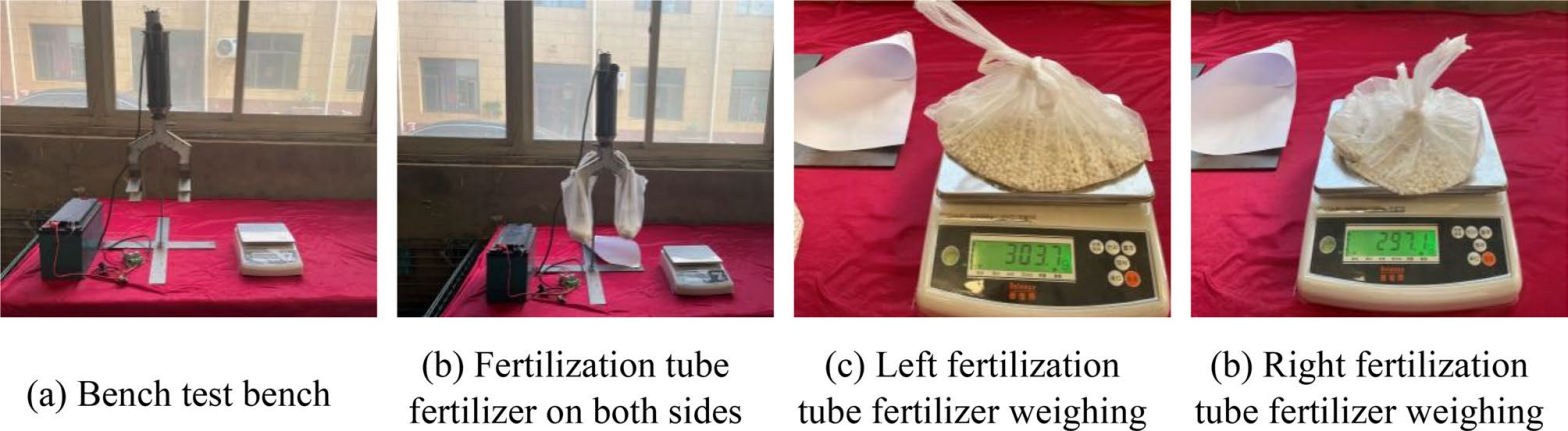
Weighing test of fertilizer amount test of fertilization pipe on both sides.

**Figure 11:**
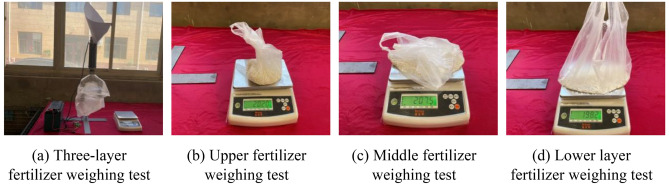
Three layers of fertilizer test weighing fertilizer device on both sides.

In order to verify the effect of static fertilization, the bench test was carried out after the fertilization device was completed. In the experiment, 600 g of compound fertilizer was taken each time, and then the compound fertilizer was introduced into the full-layer proportional fertilization device through the fertilizer tube. After the fertilizer particles were stirred evenly by the stirring structure, the fertilizer fell from the fertilizer tubes on both sides. First, the fertilizer tubes on both sides were collected by the collection device, and then the three layers of fertilizer were collected separately by the collection device. After collecting and weighing the respective weights of the fertilizer tubes on both sides, as well as the weight of the fertilizer flowing out of the three layers of fertilization port, the proportion of each layer of fertilizer was calculated. The experiment was repeated three times and the average value was obtained, as shown at Figs. 10 and 11.

### Field experiment

#### Test conditions

**Figure 12:**
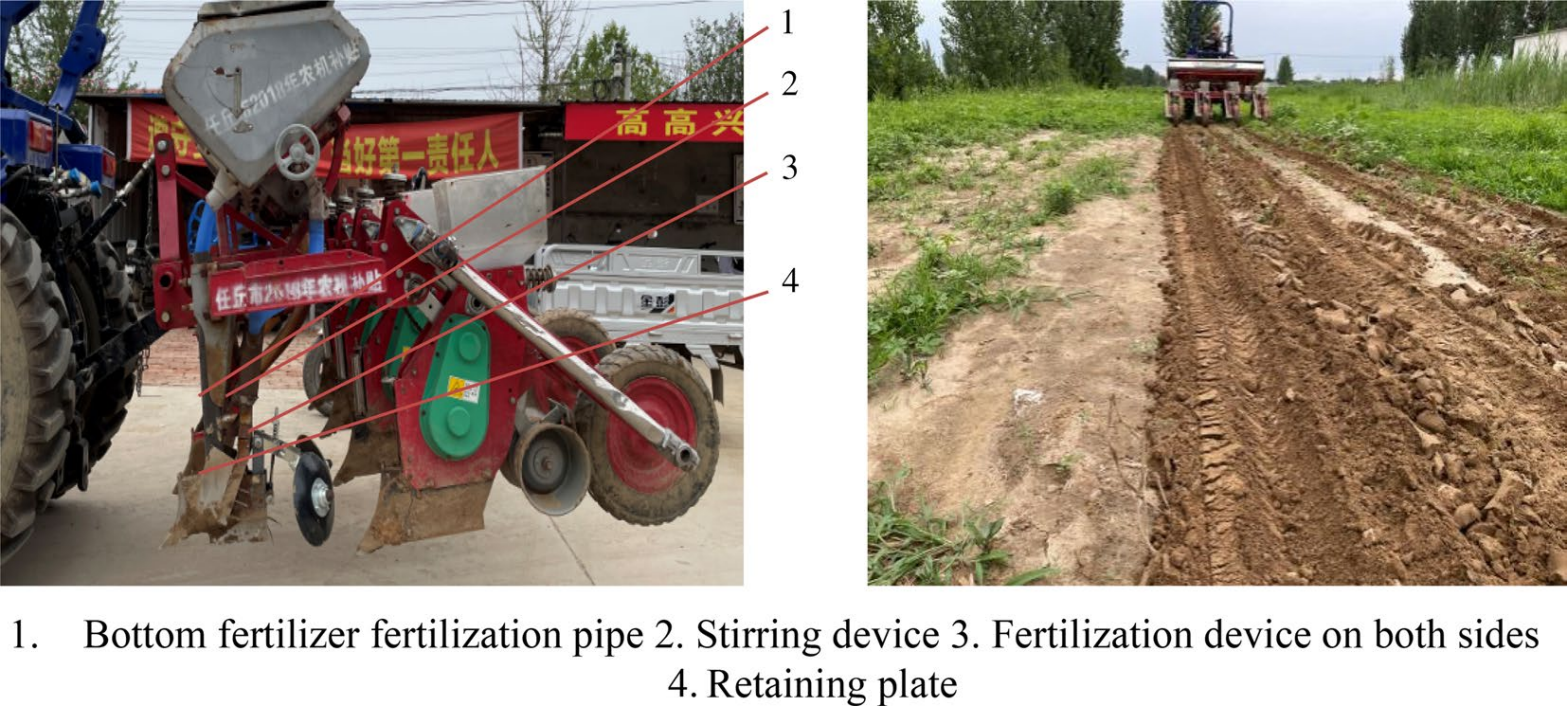
Test site.

**Figure 13:**
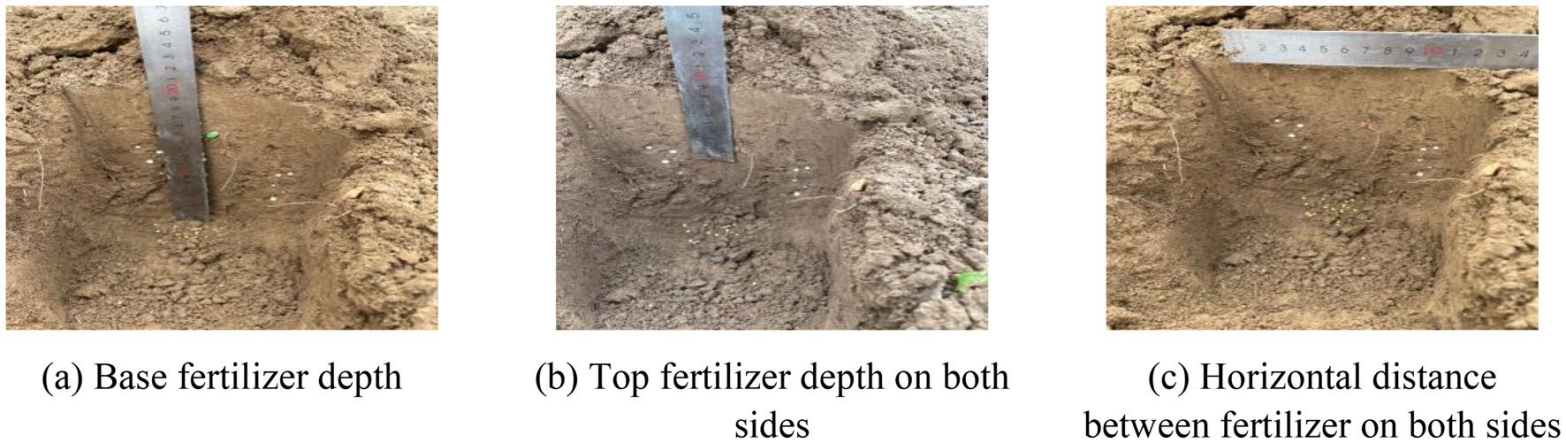
Fertilization effect diagram of U-shaped fertilization device.

In order to verify the fertilization effect of the U-shaped fertilization device, field experiments were carried out on June 17, 2021 and June 28, 2022 in Gaoyang County, Baoding City, Hebei Province. Fertilizer was Stanley compound fertilizer (N25–P_2_O_5_15–K_2_O_5_; total nutrient ≥ 45%) and Zhongxia Petrochemical stable slow-release compound fertilizer 15–15–15-Jiangyou fertilizer (N15–P_2_O_5_15–K_2_O15; total nutrient ≥ 45%), Compound fertilizer is used for conventional fertilization, and the amount of compound fertilizer is 600 kg/hm^2^. The types of U-shaped fertilization include compound fertilizer and slow-release fertilizer. The amount of compound fertilizer is 180 kg/hm^2^, and the amount of slow-release fertilizer is 420 kg/hm^2^. tractor was Lovol M1000-DA, fertilization device operating speed is 3 km/h, stirring structure stirring speed is 300 r/min. Machine forward fertilization operation 100 m, every 10 m to select a measuring point, a total of 8 measuring points, measuring U fertilization device fertilization depth and spatial distribution of full layer fertilizer, test as shown in Fig. 12. The fertilization depth is the distance from the farthest particles of the bottom fertilizer of the U-shaped fertilization device to the surface and the distance from the nearest compound fertilizer particles on both sides to the surface. Due to the soil flow during the measurement process, the soil after fertilization was cut horizontally in the fertilizer ditch (Fig. 13). The vertical distance from the bottom and top of the U-shaped fertilization to the surface and the horizontal distance between the fertilizers on both sides were measured. The average value is shown in Table 6.

**Table 6:** Measurement results of fertilization position.

Serial no.	Upper layer fertilizer (mm)	Middle layer fertilizer (mm)	Deep of lower layer fertilizer (mm)
1	197.8	88.2	90.3
2	206.0	87.3	97.6
3	203.4	89.7	98.2
4	201.3	88.5	99.4
5	201.5	90.2	92.4
6	203.0	88.3	88.5
7	202.6	92.7	90.7
8	198.4	95.2	84.3
Mean value	201.8	90.0	92.7

### Ethics Statement

The study comply with relevant institutional, national, and international guidelines and legislation.

## Results

### Stirring speed A single factor results

Through Fig. 5, it can be seen that the stirring effect is the best when the stirring speed is 200–400 r/min, and the fertilizer on both sides is consistent. Therefore, the range of single factor of stirring speed of stirring structure is 200–400 r/min.

### Bending angle B single factor result

Through Fig. 7, using the simulation software post-processing, the fertilizer output of the three fertilizer outlets of the full-layer proportional fertilization mechanism is screened at a ratio of 1:1:1 for the bending angle, and the single factor value range of the bending angle of the fertilization device is 160–170°.

### Establishment of value range of each factor of u-shaped full-layer proportional fertilization device

In summary, the main factors affecting the U-shaped full-layer proportional fertilization device are the stirring speed A of the stirring structure, the bending angle B at the fertilizer pipe of the full-layer proportional fertilization device, and the fertilization shovel operating speed C. The range of each factor: A is 200–400 r/min, B is 160–170°, C is 2–4 km/h.

### Simulation test results and analysis

#### Analysis of variance of test results and establishment of regression model

##### Analysis of variance and regression model establishment of upper fertilization

The experimental results are shown in Table 6. The Design-Expert software is used to process the experimental data, and the insignificant items are eliminated. The quadratic regression model of the upper fertilization amount of the actual fertilization device and the upper fertilization amount of the target fertilization device is obtained as follows:

13 $${\mathrm{Y}}_{1} = 203.20 + 14.00{\mathrm{A}} + 16.13{\mathrm{B}} + 12.25{\mathrm{AB}} - 10.10{\mathrm{A}}^{2} + 8.65{\mathrm{C}}^{2}$$

According to Table 6, the quadratic regression model *P* < 0.01 is extremely significant, and the lack of fit term *P* > 0.1 is significant, indicating that the fitting model can correctly reflect the relationship between various factors and errors, and can better predict the test results. A, B, AB, A^2^, B^2^ items are significant, and the remaining items are not significant. According to the regression coefficient of the model, the influence of each factor on the error is B, A, and C in descending order.

##### Analysis of variance and regression model establishment of middle fertilization

The experimental results are shown in Table 7. The Design-Expert software was used to process the experimental data, and the insignificant items were removed. The quadratic regression model of the middle-level fertilization amount of the actual fertilization device and the target fertilization device was obtained:

14 $${\mathrm{Y}}_{1} = 202.00 + 10.25{\mathrm{A}} + 16.75{\mathrm{B}} + 9.25{\mathrm{AB}} + 8.25{\mathrm{AC}} - 8.37{\mathrm{A}}^{2} + 12.12{\mathrm{C}}^{2}$$

**Table 7:** Analysis of variance of fertilization amount at upper and middle fertilizer outlets.

Projects	Source	Sum of squares	Degree of freedom	Mean square	F	*P*
Fertilization amount of upper fertilizer outlet	Model	5208.98	9	578.78	12.8	0.0014
	A	1568.00	1	1568.00	34.67	0.0006
	B	2080.12	1	2080.12	46	0.0003
	C	136.13	1	136.13	3.01	0.1263
	AB	600.25	1	600.25	13.27	0.0083
	AC	12.25	1	12.25	0.27	0.6188
	BC	16.00	1	16.00	0.35	0.5707
	A^2^	429.52	1	429.52	9.5	0.0178
	B^2^	91.04	1	91.04	2.01	0.1989
	C^2^	315.04	1	315.04	6.97	0.0335
	Residual error	316.55	7	45.22		
	Lack of fit	151.75	3	50.58	1.23	0.4089
	Pure error	164.80	4	41.20		
	Sum	5525.53	16			
Middle row fertilizer mouth fertilization amount	Model	4732.00	9	525.78	11.22	0.0022
	A	840.50	1	840.50	17.94	0.0039
	B	2244.50	1	2244.50	47.90	0.0002
	C	84.50	1	84.50	1.80	0.2212
	AB	342.25	1	342.25	7.30	0.0305
	AC	272.25	1	272.25	5.81	0.0467
	BC	42.25	1	42.25	0.90	0.3740
	A^2^	295.33	1	295.33	6.30	0.0404
	B^2^	29.01	1	29.01	0.62	0.4572
	C^2^	619.01	1	619.01	13.21	0.0083
	Residual error	328.00	7	46.86		
	Lack of fit	68.00	3	22.67	0.35	0.7934
	Pure error	260.00	4	65.00		
	Sum	5060.00	16			

*P < 0.01 is extremely significant; 0.01 ≤ *P* ≤ 0.1 for significant effect.

According to Table 5, the quadratic regression model *P* < 0.01 is extremely significant, and the lack of fit term *P* > 0.1 is not significant, indicating that the fitting model can correctly reflect the relationship between various factors and errors, and can better predict the test results. A, B, AB, AC, A^2^, C^2^, the term is significant, and the remaining terms are not significant. According to the regression coefficient of the model, the influence of each factor on the error is B, A, and C in descending order.

### Response surface analysis

When the operating speed of the fertilizer shovel is 3 km/h, the effect of the interaction between the stirring speed A of the stirring structure and the bending angle B of the fertilizer pipe on the amount of fertilizer applied to the upper fertilizer outlet is shown at Fig. 9a. When the stirring speed is constant, the bending angle of the fertilizer pipe is positively correlated with the change of the upper fertilizer output. The target of the upper fertilizer outlet is 200 g, and the optimal bending angle is 162–168°. When the bending angle of the fertilizer tube is constant, the stirring speed of the stirring structure is positively correlated with the change of the upper fertilizer output, and the better bending angle is 250–350 r/min.

When the operating speed of the fertilizer shovel is 3 km/h, the effect of the interaction between the stirring speed A of the stirring structure and the bending angle B of the fertilizer pipe on the amount of fertilizer applied to the middle layer is shown at Fig. 9b. When the stirring speed is constant, the bending angle of the fertilizer pipe is positively correlated with the change of the middle layer fertilizer output. With the middle layer fertilizer output of 200 g as the goal, the optimal bending angle is 162–168°. When the bending angle of the fertilizer pipe is constant, the stirring speed of the stirring structure is positively correlated with the change of the middle layer fertilizer output, and the optimal bending angle is 230–350 r/min.

### Parameter optimization

In order to obtain the optimal stirring speed and the bending angle of the fertilizer tube, the two regression models were optimized by using the optimization module of Design-Expert software. According to the actual working conditions and requirements of the fertilizer device, the constraint conditions of the objective function were selected to optimize the solution.

The objective function and constraint conditions are shown in Formula (15):

15 $$\left\{ {\begin{array}{*{20}c} {{\text{M}}_{1} \left( {{\text{A,B,C}}} \right) = 200\;{\text{g}}} \\ {{\text{M}}_{2} \left( {{\text{A,B,C}}} \right) = 200\;{\text{g}}} \\ {{\text{s}}.{\text{t}}.\left\{ {\begin{array}{*{20}c} {250\;{\text{r}}/{\text{min}}\; \le \;{\text{A}} \le \;350\;{\text{r}}/{\text{min}}} \\ {162^\circ \; \le \;{\text{B}}\; \le \;168^\circ } \\ {2\;{\text{km}}/{\text{h}}\; \le \;{\text{C}}\; \le \;4\;{\text{km}}/{\text{h}}} \\ \end{array} } \right.} \\ \end{array} } \right.$$

The stirring speed of the stirring structure is 300 r/min, the bending angle of the fertilizer pipe is 165°, and the professional speed of the fertilizer device is 3 km/h. The fertilizer ratio of the upper, middle and lower layers of the fertilizer pipe on both sides is 1:1:1, which meets the design requirements of the fertilizer ratio.

### Test results and analysis

#### Layered fertilization related parameters results and analysis

The results of the bench test are shown in Table 7. The error between the target fertilization ratio and the actual fertilization ratio and the variation coefficient of the stability of the upper, middle and lower layers is used as the evaluation indexes. The variation coefficient of the stability of the fertilizer discharge is obtained by Formula (16).

16 $$CV = {\frac{i}{w}} \times 100\%$$

Arranged

17 $$i = \sqrt {\frac{1}{N - 1}\sum\limits_{n = 1}^{N} {\left( {X_{n} - \frac{{\sum\limits_{n = 1}^{N} {X_{n} } }}{N}} \right)} }$$

18 $$w = \frac{{\sum\limits_{n = 1}^{N} {X_{n} } }}{N}$$

where $N$- number of tests; ${{\text{X}}_{n}}$- fertilizer rate, g; $CV$-is the coefficient of variation of fertilizer stability, %.

From Table 8, it can be seen that the average fertilization on the left and right sides of the fertilization tube is 299.5 g and 297.4 g respectively, the coefficient of variation is 0.07% and 0.08% respectively, and the relative error of the three test results on the left and right sides is within 2%, so the fertilization ratio on both sides of the fertilization tube reaches the expected 1:1 setting. The average amount of fertilizer applied on the upper, middle and lower layers of the fertilizer pipe on both sides were 200.4 g, 203.2 g and 197.7 g, respectively, and the coefficient of variation was 0.35%, 0.11% and 0.16%, respectively. The difference in coefficient of variation between each other was within 0.2%, and the relative error was within 3%, which met the requirement of 1:1:1 fertilizer at the upper, middle and lower fertilizer outlets of the expected fertilizer pipe.

**Table 8:** Fertilization ratio for each layer.

Fertilizing placement	Test results (g)			Mean value (g)	Coefficient of variation (%)	Relative error (%)
	1	2	3			
Left side	296.3	303.7	298.6	299.5	0.07	1.4
Right side	301.1	297.1	294.1	297.4	0.08	1.2
Upper ayer	201.1	202.0	199.1	200.4	0.35	0.8
Middle layer	201.0	207.5	201.2	203.2	0.11	2.1
Lower layer	197.8	198.2	197.3	197.7	0.16	0.3

### Field test results

The test shows that the top compound fertilizer applied on both sides of the U-shaped fertilization device is mainly distributed between 87.3 and 95.2 mm from the surface. The slow-controlled fertilizer discharged from the bottom of the U-shaped fertilization device is mainly distributed between 197.8 and 206.0 mm. The lateral distance of the fertilizer applied on both sides of the U-shaped fertilization device is mainly distributed between 84.3 and 99.4 mm, which is within 10 mm from the designed theoretical fertilization depth (90 mm, 200 mm, 100 mm). The distribution of fertilizer in each layer shows that there are more fertilizers in the lower layer and less fertilizers in the upper and middle layers, which achieves the expected effect.

### Effects of different fertilization methods on physical characteristics and yield of corn plants

**Figure 14:**
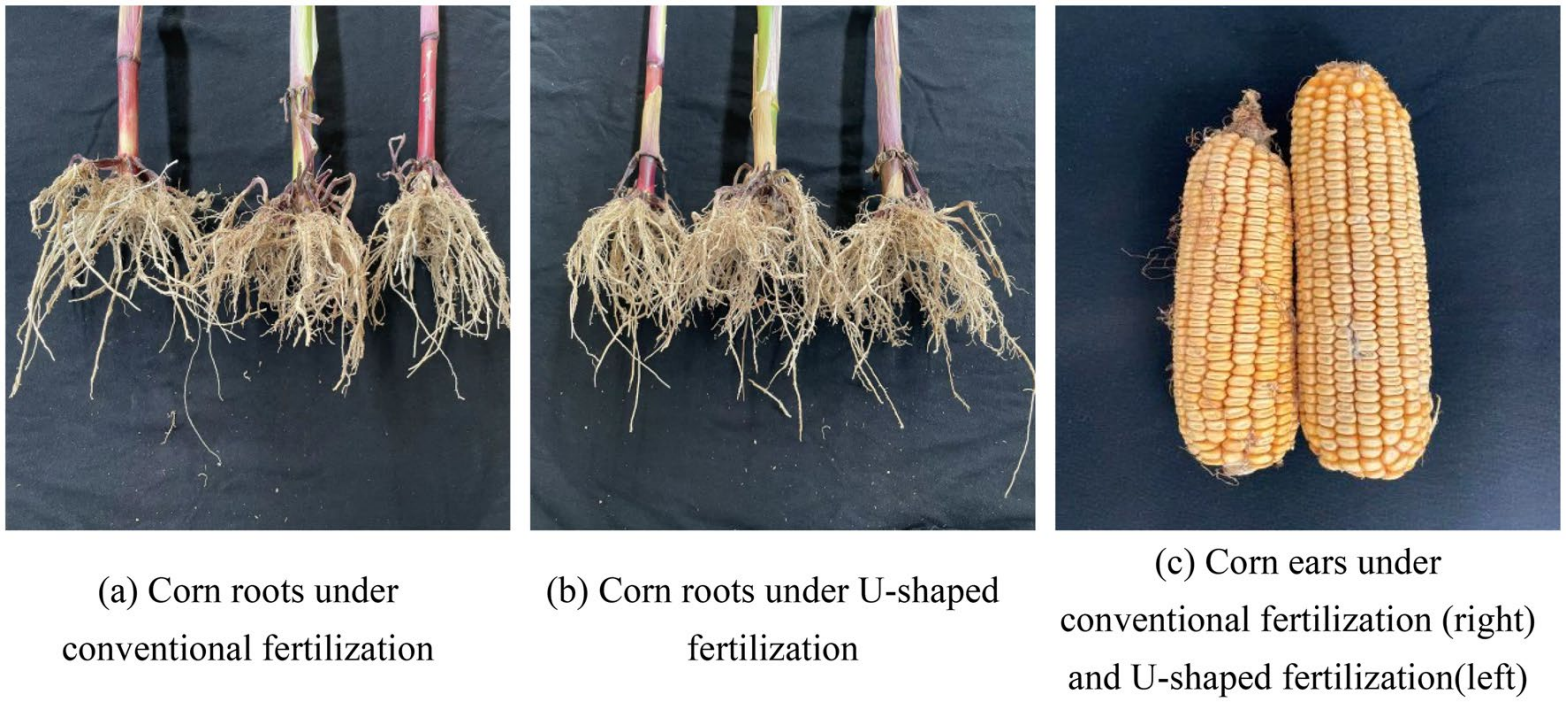
Comparison of corn root and ear under traditional fertilization and U-shaped fertilization.

In the experimental field of Gaoyang County, Baoding City, Hebei Province, for the two years of summer corn from 2021 to 2022, the conventional fertilization method and the designed U-shaped fertilization method was used to sample and compare the plant height, stem diameter, dry weight, root number, yield and other parameters of summer corn at maturity. Each year, corn grown at maturity was randomly selected from the experimental fields of different fertilization methods for parameter measurement and recording, and finally presented in the form of average values of corn parameters, as shown in Table 9. Through the comparison of the parameters in the table, it can be seen that the U-shaped fertilization method has a promoting effect on the growth of summer corn compared with the conventional fertilization method, especially in the 100-grain weight and yield. The comparison of roots and ears of corn under conventional fertilization and U-shaped fertilization are shown in Fig. 14.

**Table 9:** Interaction of different fertilization methods on growth and yield traits of summer corn at maturity.

Year	Treatment	Plant height (cm)	Dry matter accumulation (g/plant)	Stem diameter (cm)	Number of roots (piece/plant)	Root length (mm)	100-grain weight (g)	Yield (kg/hm^2^)
2021	CF	281.43	263.14	7.80	49.00	153.00	33.41	13,215.13
	UF	283.14	282.57	7.80	55.00	184.57	35.74	14,522.22
2022	CF	281.00	269.14	7.94	49.00	163.43	32.24	13,239.50
	UF	282.14	295.29	7.89	56.00	197.43	36.29	15,202.28
Year		ns	**	**	ns	ns	ns	ns
Treatment		ns	**	ns	**	**	**	**
Year × Treatment		ns	ns	ns	ns	ns	**	ns

CF is conventional fertilization treatment; UF represents U-shaped fertilization treatment.

ns indicates no significant difference;

*Indicates that there is a significant difference at the level of 0.05; ** indicates that there is a significant difference at the level of 0.01.

## Conclusion

1. An U-shaped fertilization device is designed, which is mainly composed of stirring structure, fertilizer guide piece, fertilizer piece, etc. The diameter of the designed stirring shaft is 16 mm, the inclination angle a of the retaining plate is determined, and the force of the retaining plate is also established.
2. The structural parameters of the U-shaped fertilization device were optimized by quadratic regression orthogonal rotation combination designs. When the stirring speed of the stirring structure was 300 r/min, the bending angle of the fertilization tube was 165° and the operating speed of the fertilization device was 3 km/h, the U-shaped fertilization effect was better.
3. The bench test shows that the error of the fertilizer application on both sides is 1.4% and 1.2% respectively compared with the simulation results, and the coefficient of variation is not more than 0.1%. Compared with the simulation results, the errors of fertilization amount of each layer were 0.8%, 2.1% and 0.3% respectively, and the coefficient of variation was not more than 0.4%. The field experiment shows that the error between the distribution depth of fertilizer in soil and the theoretical design is within 10 mm, and the error between the distribution of fertilizer on both sides and the theoretical design is within 10 mm, which meets the design requirements.
4. The average number of corn roots after U-shaped fertilization is 55–56, the average root length was 184.57–197.43 mm, and the average yield increased by 1000–2000 kg/hm^2^. Compared with the corn after traditional side fertilization, it increased by 12.2–14.3%, 20.6–20.8%, 9.9–14.8%, respectively.
5. Full-layer proportional fertilization technology is mainly to apply the fertilizer required during the growth period of corn to the soil in a one-time proportional manner, which not only improves the utilization rate of fertilizer, but also reduces the process of staged fertilization operation, achieves cost savings and efficiency, and also conforms to the ' double reduction ' policy formulated by the Ministry of Agriculture and Rural Affairs^23^, that is, reducing the use of fertilizer and agricultural use to reduce environmental pollution. However, this device (U-shaped fertilization device) is only used in the experimental field (sandy soil). To promote it nationwide, it is necessary to make relevant improvements to the key parts of the device to adapt to different soil requirements.

## Supplementary Information

Supplementary Information.

## Data Availability

All data generated or analysed during this study are included in this published article [and its supplementary information files].
