# Supplementary material for: Design optimization and experiment of corn U-shaped fertilization device
Source: Sci Rep. 2023 Jun 14;13:9669. doi: 10.1038/s41598-023-36746-5 (PMC10267109; doi:10.1038/s41598-023-36746-5)
Supplement: Supplementary file 1 — Supplementary Information. [file 41598_2023_36746_MOESM1_ESM.docx]

The red part is the original data

| 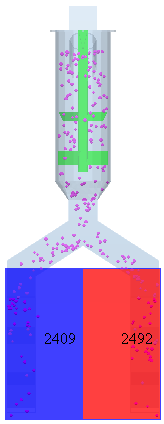 | 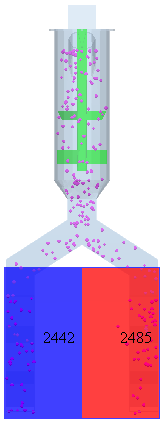 | 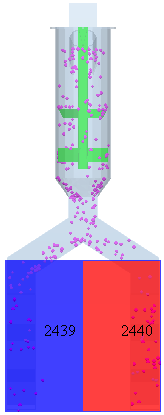 | 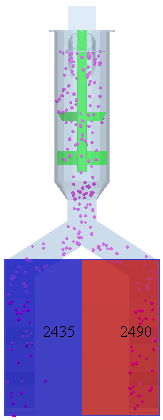 | 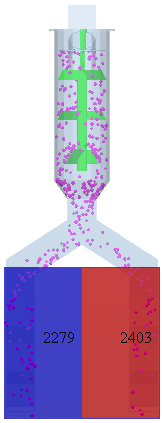 |
| --- | --- | --- | --- | --- |
| （a）A=100  r/min | （b）A=200 r/min | （c）A=300 r/min | （d）A=400 r/min | （e）A=500  r/min |
| Figure 5. Detection effect of stirring structure | | | | |

Q1:

Answer: In this simulation, each speed is done three times, presenting the most ideal group in the table. The original data of the left and right fertilizer tubes are

1. A=100r/min：2387,2450; 2397,2488; 2409,2492
2. A=200r/min：2442,2485; 2441,2501; 2443,2489
3. A=300r/min: 2439,2440; 2433,2444; 2441,2459
4. A=400r/min: 2435,2490; 2421,2497; 2437,2524
5. A=500r/min: 2279,2403; 2173,2411; 2377,2487

Q2:

| 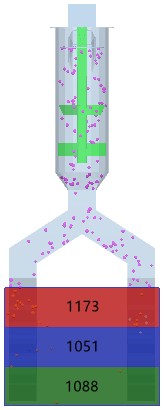 | 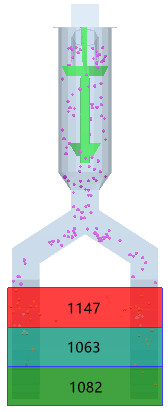 | 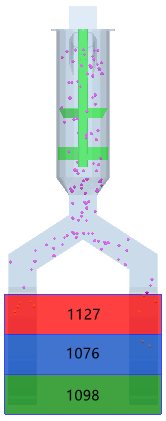 | 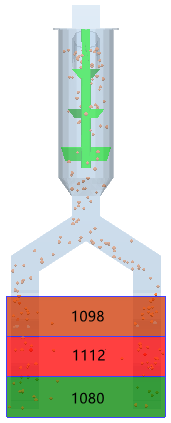 |
| --- | --- | --- | --- |
| （a）B=140° | （b）B=150° | （c）B=160° | （d）B=170° |
| Figure 7. Fertilization amount at different bending angles | | | |

Answer: Because the optimum stirring speed obtained by the single factor test of stirring speed is 300 r/min, the stirring speed is 300 r/min. The number of tests is not limited. The original data of the upper and lower layers of the fertilization tube are as follows.

1. B=140°：1173,1051,1088; 1153,997,1001;1187,1027,1097; 1056,1032,1103
2. B=150°：1149,1057,1077; 1147,1063,1082; 1137,1068,1055
3. B=160°：1127,1076,1098; 1033,1097,1079;
4. B=170°：1098,1112,1080; 1076,1099,1127; 1097,1127,1073

Q3:

Table 5 Test plan and results

| Numbering | Stirring speed A / (r/min) | Folding angle  B / (°) | Operating speed C / (km/h) | Upper fertilizer output  M_1_ / kg | Middle fertilizer amount  M_2_ / kg |
| --- | --- | --- | --- | --- | --- |
| 1 | 400.00 | 170.00 | 3.00 | 180 | 179 |
| 2 | 200.00 | 170.00 | 3.00 | 188 | 184 |
| 3 | 400.00 | 160.00 | 3.00 | 183 | 190 |
| 4 | 200.00 | 160.00 | 3.00 | 240 | 232 |
| 5 | 400.00 | 165.00 | 2.00 | 193 | 210 |
| 6 | 200.00 | 165.00 | 2.00 | 213 | 211 |
| 7 | 400.00 | 165.00 | 4.00 | 187 | 184 |
| 8 | 200.00 | 165.00 | 4.00 | 214 | 218 |
| 9 | 300.00 | 170.00 | 2.00 | 207 | 203 |
| 10 | 300.00 | 160.00 | 2.00 | 240 | 234 |
| 11 | 300.00 | 170.00 | 4.00 | 189 | 193 |
| 12 | 300.00 | 160.00 | 4.00 | 230 | 237 |
| 13 | 300.00 | 165.00 | 3.00 | 203 | 199 |
| 14 | 300.00 | 165.00 | 3.00 | 197 | 202 |
| 15 | 300.00 | 165.00 | 3.00 | 213 | 215 |
| 16 | 300.00 | 165.00 | 3.00 | 205 | 201 |
| 17 | 300.00 | 165.00 | 3.00 | 198 | 193 |

Answer：In the simulation model, the corresponding influencing factors are used for simulation test and stirring speed, bending angle and working speed. In the post-processing of the simulation software, the counter is used to count the amount of fertilizer in the upper and middle layers. The test is repeated three times, and the best one is taken for test analysis. The original data of the three tests are :

| Upper fertilizer output  M_1_ / kg | Middle fertilizer amount  M_2_ / kg |
| --- | --- |
| 197; 175; 180 | 187, 193, 179 |
| 170, 189, 188 | 179, 180, 184 |
| 183, 189, 193 | 190, 199, 197 |
| 240, 210, 224 | 212, 210, 232 |
| 187, 179, 193 | 197, 213, 210 |
| 223, 213, 203 | 197, 211, 199 |
| 199, 187, 174 | 176, 180, 184 |
| 207, 214, 203 | 218, 200, 197 |
| 207, 209, 211 | 191, 203, 220 |
| 212, 214, 240 | 234, 221, 201 |
| 189, 199, 176 | 220, 193, 221 |
| 221, 209, 230 | 237, 201, 199 |
| 190, 214, 203 | 199, 179, 211 |
| 210, 197, 191 | 200, 202, 183 |
| 213, 200, 197 | 215, 230, 180 |
| 210, 205, 193 | 230, 201, 202 |
| 198, 187, 210 | 193, 230, 220 |

Q4: Raw data of fertilization location measurement results

| Serial No. | Upper layer Fertilizer  /mm | Middle layer Fertilizer  /mm | Deep of lower layer fertilizer  /mm |
| --- | --- | --- | --- |
| 1 | 197.8 | 88.2 | 90.3 |
| 2 | 206.0 | 87.3 | 70.3 |
| 3 | 203.4 | 89.7 | 68.7 |
| 4 | 201.3 | 88.5 | 99.4 |
| 5 | 201.5 | 90.2 | 92.4 |
| 6 | 203.0 | 88.3 | 88.5 |
| 7 | 202.6 | 92.7 | 90.7 |
| 8  9  10  11  12 | 198.4  180.5  173.8  188.3 | 95.2  120.3  117.5 | 84.3  97.6  98.2  11.2  80.1 |
| mean value | 196.05 | 95.79 | 80.98 |

Q5：Table 9 The interaction effect of different fertilization methods on the growth and yield traits of summer maize analysis of the original data

| 年度  Year | 处理  Treatment | 株高  Plant height（cm） | 干重  Dry matter accumulation（g/plant） | 茎粗  Stem diameter（cm） | 根系数量  number of roots  (piece) | 根系长度  root length  (mm) | 百粒重  100-grain weight  （g） | 产量  Yield（kg/hm2） |
| --- | --- | --- | --- | --- | --- | --- | --- | --- |
| 2021 | CF | 281.43 | 263.14 | 7.80 | 49.00 | 153.00 | 33.41 | 13215.13 |
|  | UF | 283.14 | 282.57 | 7.80 | 55.29 | 184.57 | 35.74 | 14522.22 |
| 2022 | CF | 281.00 | 269.14 | 7.94 | 48.57 | 163.43 | 32.24 | 13239.50 |
|  | UF | 282.14 | 295.29 | 7.89 | 55.71 | 197.43 | 36.29 | 15202.28 |
| Year | | ns | ** | ** | ns | ns | ns | ** |
| Treatment | | ns | ** | ns | ** | ** | ** | ** |
| Year×Treatment | | ns | ns | ns | ns | ns | ** | ** |

The corresponding original data

| Year | Treatment | Plant height（cm） | Dry matter accumulation（g/plant） | Stem diameter（cm） | number of roots  (piece) | root length  (mm) | 100-grain weight  （g） | Yield（kg/hm2） |
| --- | --- | --- | --- | --- | --- | --- | --- | --- |
| 2021 | CF | 287.33  274.90  275.38  288.11 | 259.42  263.54  259.76  269.84 | 7.81  7.63  7.78  7.98 | 50.00  48.00  49.00 | 154.22  149.56  155.22 | 32.51  34.56  33.16 | 13275.87  13154.39 |
|  | UF | 286.14  275.64  287.22  283.56 | 287.87  277.89  284.67  279.85 | 7.84  7.81  7.73  7.82 | 53.00  58.00  54.00 | 187.87  186.54  179.30 | 36.37  35.29  35.56 | 14514.38  14530.06 |
| 2022 | CF | 283.18  279.36  284.59  276.87 | 270.34  267.54  270.27  268.41 | 7.99  7.83  7.87  8.07 | 49.00  51.00  47.00 | 167.53  162.98  159.78 | 32.37  32.79  31.56 | 13225.43  13253.57 |
|  | UF | 283.99  283.78  283.27  277.52 | 293.79  289.56  298.45  299.36 | 7.53  8.21  7.77  8.05 | 57.00  56.00  55.00 | 199.53  189.56  203.2 | 35.49  36.78  36.60 | 15236.41  15168.15 |
